# Film education and art therapy for mental health in college students: a systematic review

**DOI:** 10.3389/fpsyg.2026.1749029

**Published:** 2026-04-17

**Authors:** Mali Liang, Xinlei Ji

**Affiliations:** 1 School of Teacher Development, Chongqing University of Education, Chongqing, China; 2 School of Literature, Tianjin Normal University, Tianjin, China

**Keywords:** art therapy, college students, film, mental health, psychological resilience

## Abstract

Mental health issues are becoming increasingly prominent among college students, and psychological resilience is a crucial factor for individuals to maintain a good mental state when facing pressure and challenges. Art therapy, especially when combined with film production, offers college students a unique way to express themselves and to regulate their emotions. This systematic review aimed to explore the application value and effects of film education combined with art therapy in promoting mental health and psychological resilience among college students. A comprehensive literature search was conducted in PubMed from 2020 to 2025 using the keywords “art therapy,” “film,” and “mental health.” Conference proceedings, comments, and non-subject-related literature were excluded. This study was performed and reported in strict accordance with the Preferred Reporting Items for Systematic Reviews and Meta-Analyses (PRISMA) 2020 guidelines. A total of 128 records were initially identified, and 28 eligible studies were included after screening. The results demonstrate that integrated film education and art therapy interventions effectively support emotional expression, stress relief, meaning-making, and social communication, and contribute to the improvement of psychological resilience and mental health status in college students. This review also identifies key research gaps and provides directions for future interdisciplinary research. The findings support the implementation of film-based creative and expressive arts interventions in mental health education for university students.

## 1 Introduction

College students’ mental health is defined as a state of positive psychological functioning wherein individuals demonstrate sound interpersonal adaptation, effective coping capacities with academic, interpersonal, and developmental stressors, and achieve healthy development across cognitive, emotional, and social domains (Zhang S. et al., 2024). A critical protective factor underlying this state is psychological resilience, which (Sisto et al., 2019a; Swami et al., 2024a) conceptualize as a dynamic psychological capacity enabling college students to recover from adversity, stress, and negative emotions while sustaining positive adaptation and psychological growth through the mobilization of internal psychological resources and external social support. Film education, as a comprehensive educational approach centered on film as the core medium, encompasses diverse components including film appreciation, filmmaking practice, and film criticism (Zhang et al., 2025a; Lorint, 2022b). Beyond imparting film-related knowledge and technical skills, its core objectives include enhancing students’ esthetic literacy, fostering emotional expression capabilities, and promoting psychological wellbeing. Complementarily, art therapy is a evidence-based psychological intervention that utilizes various artistic forms as therapeutic media (Malchiodi, 2023a; Hu et al., 2021a). It guides individuals to express suppressed emotions, explore self-identity, and regulate psychological states through creative processes and artistic appreciation, making it highly applicable to mental health education and targeted interventions for college student populations.

Art therapy uses the creative process of art to promote psychological, emotional, and mental health (Sanjula and Sathyamurthi, 2026a). This enables individuals to express personal emotions and feelings, reduce stress, enhance self-health awareness, and develop coping skills (Malchiodi, 2023a). Art therapy is not limited to painting; it also includes various expressive art therapy forms, such as sculpture, music, dance, drama, and writing (Chiang et al., 2019; Hu et al., 2021b; Van Lith and Ettenberger, 2023; Bokoch et al., 2025). For instance, art therapy through painting can reflect an individual’s psychological state through colors and lines, facilitating doctors’ assessment and treatment (Bokoch et al., 2025). Expressive art therapy can improve the mental health of college students and their satisfaction with classes (Guangli, 2025a). In the educational field, art therapy is widely applied to improve students’ mental health, especially among college students facing academic pressure, interpersonal problems, and anxiety regarding future planning (Guangli, 2025a). Public art education can enhance the mental health of college students (Zhang S. et al., 2024). The combination of film education and art therapy as a form of creative art provides college students with a multidimensional expression platform (Zhang et al., 2025b). Through participation in the conception, scriptwriting, shooting, and editing of films, students can actively explore and express their emotions, thoughts, and experiences (Goodwin et al., 2021; Cui and Wang, 2022). This process of active participation and creative expression helps enhance self-efficacy and psychological resilience (Swami et al., 2024a). Moreover, film production can promote teamwork and communication skills, strengthen students’ social support networks, and further improve their mental health (Popova and Nosek, 2025a). Existing research shows that film education and the combination of film and art therapy have the potential to improve the mental health of college students (Lorint, 2022a). Film and television production provide a safe and creative space for students to express and explore their emotions (Krishnasamy and Van Lith, 2025a). By writing scripts and performing them, they can better understand and manage their emotions (Du et al., 2022). Simultaneously, completing a film project can significantly enhance students’ self-efficacy (Jin and Ye, 2022). From project planning to final completion, the success of each stage strengthens their confidence and sense of achievement (Swami et al., 2024a). Film production usually requires teamwork, which helps students build and strengthen their social support network (Black et al., 2025a). During the joint completion of the project, they can learn how to cooperate with others and communicate with and support each other (Du et al., 2022). Various challenges, such as technical issues, budget constraints, and time management, may be encountered during the film production process. Solving these problems can cultivate students’ creative thinking and problem-solving abilities (Bi et al., 2024). Creative artistic activities can help students relieve themselves from academic pressure and provide a way to relax and entertain themselves (Martin et al., 2018; Bi et al., 2024). Film as a medium has been used in adolescent mental health education, but its application in the university student population still requires further exploration (Gagliano et al., 2023).

Artistic expression is a natural way of emotional release and psychological communication, and film, as a comprehensive expressive art form integrating image, sound and narrative, expands the expression carrier of traditional expressive arts therapy (Malchiodi, 2023b; Sanjula and Sathyamurthi, 2026b). Film education and production allow college students to express unconscious emotions and psychological needs that are difficult to verbalize through script creation, lens expression and video editing, realizing the therapeutic effect of emotional catharsis and psychological adjustment. College students can construct their own life narratives and visual stories through film creation, and re-examine their life experiences, emotional conflicts and cognitive biases in the storytelling process, thus realizing identity construction and meaning-making (Goodwin et al., 2021; Krishnasamy and Van Lith, 2025b; Tripon, 2024). Meanwhile, watching and discussing psychological films can also help students understand the life narratives of others, evoke emotional resonance and develop empathy, which further enriches the therapeutic connotation of film education. Psychological resilience is a dynamic process of individuals’ positive adaptation to stress and adversity, and film education integrated with art therapy acts on the three core links of college students’ stress coping (cognitive appraisal, coping strategy selection, and coping outcome feedback) (Sisto et al., 2019a; Swami et al., 2024b). Film production and appreciation can help students build positive cognitive appraisal of stress, master diversified emotional regulation and stress coping strategies, and enhance their sense of self-efficacy and social support, thus promoting the development of psychological resilience and improving their ability to resist mental health risks.

In the present study, film education is conceptualized as a hybrid model integrating pedagogical practice with therapeutic intervention, specifically tailored to address the mental health needs of college students. This model is characterized by two core, mutually reinforcing attributes: First, its pedagogical practice attribute: Grounded in the fundamental tenets of higher education, film education takes curriculum as the primary vehicle, systematically integrating film knowledge transmission, esthetic cultivation, and mental health education into the instructional process (Guangli, 2025b; Zhang and Zhao, 2024a). As an integral component of university public art education systems, it aims to foster students’ comprehensive competencies encompassing esthetic literacy, critical thinking, and psychological wellbeing through intentional, evidence-informed instructional design. Second, its therapeutic intervention attribute: Building on this pedagogical framework, film education incorporates core methodologies of art therapy, leveraging film appreciation and production as targeted psychological intervention modalities (Krishnasamy and Van Lith, 2025a; Du et al., 2022). Centered on the mental health needs of college students, this attribute facilitates therapeutic outcomes including effective emotional regulation, stress mitigation, and enhanced psychological resilience via creative expression and narrative engagement. In essence, it functions as a purposeful, contextually adaptive mental health intervention tailored to the developmental characteristics of young adults.

## 2 Materials and methods

This systematic review was conducted and reported in full compliance with the Preferred Reporting Items for Systematic Reviews and Meta-Analyses (PRISMA) 2020 guidelines.^1^ The completed PRISMA 2020 checklist (Supplementary File 1) and flow diagram (Supplementary File 2) are included as Supplementary material to ensure transparency, standardization, and reproducibility of the entire research process.

### 2.1 Literature search and screening

A comprehensive literature search was performed in Pubmed databases using predefined search terms and retrieval strategies focused on art therapy, film education, and mental health among college students. Duplicate records were removed automatically and manually. Two researchers independently screened the titles, abstracts, and full texts based on preset inclusion and exclusion criteria. Any discrepancies during the screening process were resolved through discussion or by consulting a third senior researcher.

### 2.2 Data extraction

A standardized data extraction form was used to extract key information from the finally included studies, including basic study information, research design, participant characteristics, intervention measures, outcome indicators, and core conclusions. All extracted data were cross-verified to ensure accuracy, completeness, and consistency.

### 2.3 Quality assessment

Two researchers independently evaluated the methodological quality and risk of bias of the included studies using appropriate quality assessment tools. The evaluation results were summarized systematically to ensure the scientificity and reliability of the synthetic conclusions.

### 2.4 Software applications and their value under PRISMA guidelines

Three professional software tools were applied to support the implementation and standardized reporting of this systematic review in full compliance with the PRISMA 2020 framework. The seminal research of CiteSpace laid the foundation for the field of bibliometrics (Chen, 2006). CiteSpace was used to perform visual analyses of literature, including keyword co-occurrence, research hotspot mining, and trend evolution analysis, which helped to realize objective and comprehensive identification and classification of literature, enhancing the transparency and systematicness of literature screening. In recent years, it has been widely used in research on art therapy and mental health, such as Xing et al. (2024) who used this tool to analyze the differences in research hotspots of art therapy in education between China and foreign countries. Hiplot provides an efficient tool for scientific data visualization (Li H. et al., 2021). Hiplot was adopted for quantitative sorting, statistical analysis and visual processing of extracted data, which supported standardized data synthesis and result display, and improved the accuracy and repeatability of data analysis required by PRISMA. Recently, Bi et al. (2024) used it to complete the statistical analysis and visualization of intervention effects in a study on digital health interventions for college students. As a professional scientific drawing tool, BioRender has become a common choice for drawing figures in international journals (Rajkomar et al., 2020). BioRender was utilized to draw standard visual illustrations, including the flow diagram and research framework diagram, which ensured the standardized presentation of research procedures and results, and met the transparent reporting requirements of the PRISMA guidelines. These software tools jointly supported the whole process of literature identification, screening, data management, statistical analysis and standardized visualization, effectively ensuring the scientificity, standardization and transparency of this systematic review under the PRISMA framework. Bokoch et al. (2025) used this tool to draw an intervention mechanism framework diagram in cross-research on art therapy and neuroscience.

## 3 Results

**FIGURE 1:**
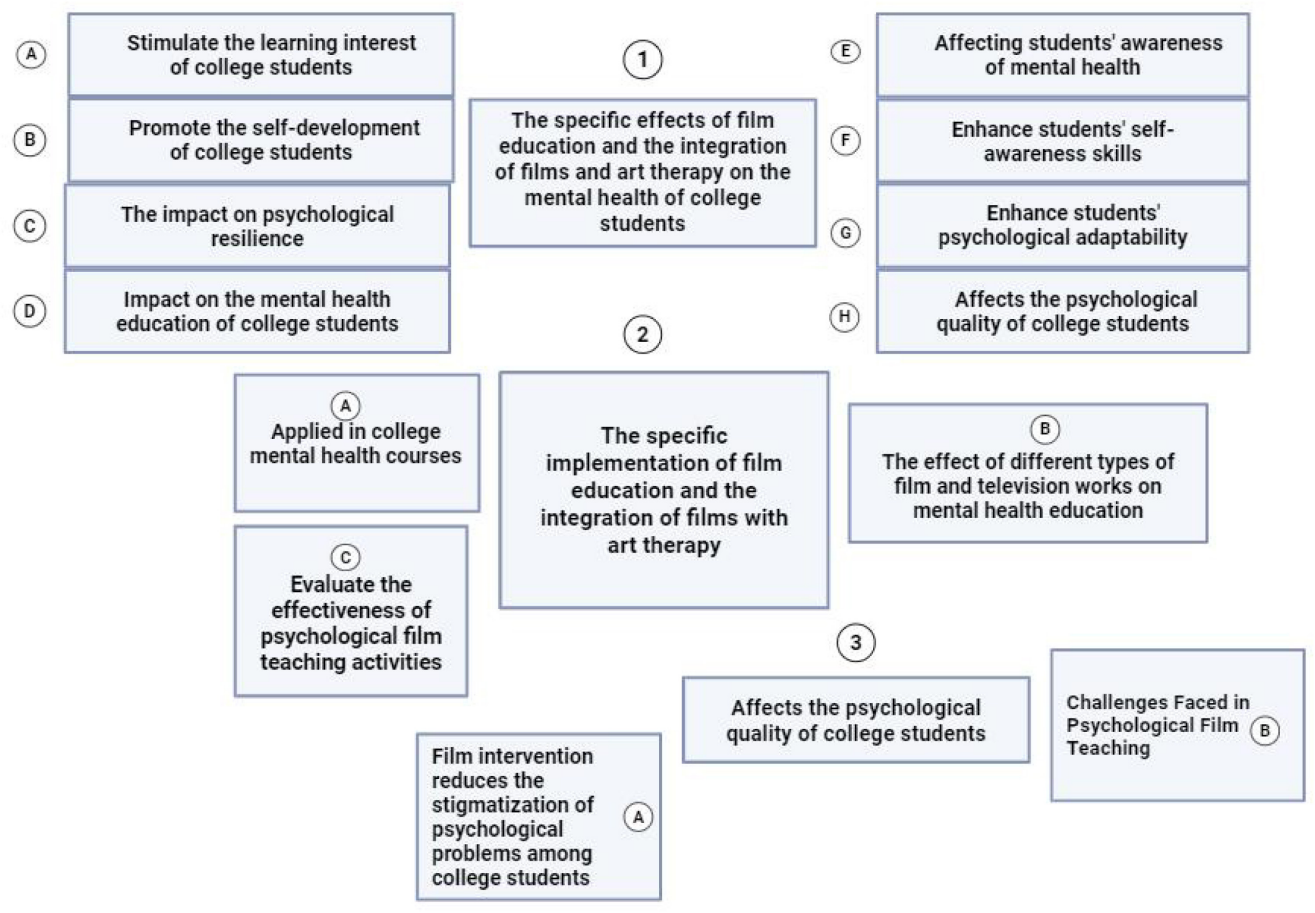
Conceptual framework of film education integrated with art therapy for college students’ mental health. This figure organizes the research into three core thematic dimensions: (1) The specific effects of film education and art therapy on college students’ mental health, this dimension categorizes the positive outcomes of the intervention; (2) The specific implementation of film education integrated with art therapy, this dimension outlines the practical application of the intervention; and (3) The impact of the integrated intervention on the psychological quality of college students. This dimension focuses on the broader outcomes for students, linking the intervention’s effects to improvements in their overall psychological wellbeing and quality of life. Figure created in BioRender.com.

To visually present the theoretical logic and research design of this study, Figure 1 illustrates the conceptual framework of film education integrated with art therapy for college students’ mental health. Additionally, Supplementary Table 1 summarizes the basic characteristics of the 28 included studies (including research design, sample size, intervention duration, etc.). This framework structures the entire study around three core thematic dimensions, covering the positive intervention effects of film education and art therapy on college students’ mental health, the practical implementation pathways of the integrated educational model, and its comprehensive influence on students’ psychological quality. By systematically connecting intervention practices, psychological effects, and mental health promotion, the framework clearly demonstrates the value and operational logic of applying art-integrated film education in improving college students’ psychological wellbeing and quality of life.

### 3.1 The specific effects of film education and the integration of films and art therapy on the mental health of college students

Art interventions involve integrating artistic activities into mental health education courses. Through various artistic activities, such as art appreciation, creation, and discussion, students’ personalities can be improved and their psychological qualities can be enhanced (Gaiha et al., 2021; Liu et al., 2024). College students can use the artistic creation process as a psychological adjustment tool to help them cope with emotional issues such as stress, anxiety, and depression; build a positive social support network; and improve social adaptability, thereby promoting the all-round development of their psychological qualities (Jin and Ye, 2022; Wu et al., 2025; Zhang X. et al., 2024; Liu et al., 2024). The application prospects of film education and film production etc., art therapy in college students’ mental health education will be even broader, and the integration of film education and art therapy into the curriculum will have numerous positive effects on their mental health of college students. Through the appreciation of psychological films, college students can acquire knowledge about mental health, enhance their learning enthusiasm, understand abstract psychological concepts, evoke emotional resonance, develop empathy, learn to view problems from different perspectives, reflect on their values and life choices, thereby better understanding themselves, promote emotional expression and cognitive reconstruction, learn different problem-solving strategies, cultivate social responsibility and civic consciousness, obtain psychological comfort, establish good interpersonal relationships, strengthen the sense of identity and belonging to one’s own culture, thereby improving mental health levels, receive more social support, and achieve positive impacts on emotional, cognitive, and social aspects (Goodwin et al., 2021; Krishnasamy and Van Lith, 2025a). Art therapy enables individuals to express their emotions in a safe and supportive environment. Art therapy can help individuals learn new coping strategies, enhance their ability to cope with stress, and handle life challenges better. Moreover, in counseling and therapy, films can be used as an introduction to guide students to engage in artistic creation, thereby providing a deeper understanding of their inner worlds and formulating more effective treatment plans (Geller, 2020; Shukla et al., 2022).

#### 3.1.1 Stimulate the learning interest of college students

Psychological film teaching can stimulate college students’ learning interests in various ways, including content selection, teaching methods, and interaction design. Public art education indirectly promotes mental health by enhancing students’ esthetic and cultural literacy, thereby increasing their learning interest (5). Films and television can be combined with classroom teaching, psychological counseling, community activities, etc., to form a more comprehensive mental health education system (Zhang and Zhao, 2024b; Hu et al., 2022).

#### 3.1.2 Promote the self-development of college students

Psychological films, as educational tools, can help college students understand the importance of mental health and evoke emotional resonance. For instance, excellent narrative films often enable viewers to reflect deeply on human nature and social issues. However, their effectiveness in mental health education requires further exploration (Goodwin et al., 2021; Krishnasamy and Van Lith, 2025a). Additionally, integrating psychological film teaching with other psychological education methods, such as psychological lectures, psychological counseling, and group counseling, can promote the self-development of college students more comprehensively through the combination of various educational approaches.

#### 3.1.3 Enhance students’ self-awareness skills

Psychological film teaching enhances self-awareness through various means such as the dissemination of psychological knowledge and promotion of students’ self-development (Fan and Cui, 2024). Besides psychological film teaching, other art therapies, such as painting art therapy and expressive art therapy, are also applied in mental health education to improve students’ self-awareness (Van Lith and Ettenberger, 2023). Expressive art therapy has broad development prospects in the field of group counseling for college students’ mental health education (Xing et al., 2024). A 4-month intervention study compared the experimental group (1,334 students) and the control group (1,139 students) and showed that public art education promotes the psychological health of college students (Zhang and Zhao, 2024b). Teachers can integrate art interventions into university mental health education through diverse artistic activities, effectively improving students’ personalities and enhancing their psychological quality of college students (Zhang and Zhao, 2024b; Liu et al., 2024).

#### 3.1.4 The impact on psychological resilience

Psychological resilience refers to an individual’s ability to adapt positively and recover when facing stress, challenges, or adversity (Sisto et al., 2019b; Swami et al., 2024b). From a cognitive perspective, film and television production can enhance students’ problem-solving skills and strengthen their cultivation of critical thinking, thereby enabling them to better understand the world and society. It can also enhance their self-awareness and emotional management abilities, boost students’ self-confidence, enable them to more actively confront challenges and difficulties, enhance students’ interpersonal communication skills, cultivate students’ sense of social responsibility, have a positive impact on the psychological resilience of college students, and improve teamwork skills, which is also an important factor for success in the future workplace (Zhang and Zhao, 2024b; Goodwin et al., 2021).

#### 3.1.5 Affecting the psychological quality of college students

Film and television content can influence the psychological qualities of college students through various mechanisms (Goodwin et al., 2021; Krishnasamy and Van Lith, 2025b). Art education can significantly enhance the mental health literacy of college students. A study conducted among Chinese college students showed that students in the experimental group who received public art education significantly outperformed those in the control group in terms of their mental health knowledge (Zhang and Zhao, 2024b). Moreover, cognitive behavioral art therapy, which integrates core values, can help students identify and adjust unhealthy cognitive and emotional patterns (Cui and Wang, 2022). Research has shown that art therapy can effectively alleviate the psychological stress experienced by students with traumatic stress disorder (Wang et al., 2025). Expressive art therapy can significantly improve the mental health and classroom satisfaction of college students (Shukla et al., 2022). Art therapy, an effective psychological intervention method, plays an important role in the healthy development of college students (Hu et al., 2021b; Wang et al., 2025).

#### 3.1.6 Impact on the mental health education of college students

Film and television works influence the mental health education of teenagers through various mechanisms, such as providing mental health knowledge, promoting emotional resonance, and triggering thinking and discussions. Films and television works can serve as auxiliary tools for mental health education, but they cannot replace other educational methods. They can be combined with classroom teaching, psychological counseling, community activities, etc., to form a more comprehensive mental health education system (Goodwin et al., 2021; Tripon, 2024). Families play an important role in college students’ media usage (Procentese et al., 2019; Wu et al., 2025). The participation and attention of parents can help them use the media more healthily, thereby promoting mental health.

#### 3.1.7 Affecting students’ awareness of mental health

Psychological film teaching influences students’ mental health awareness through various means, popularizes psychological knowledge, enhances mental health literacy, and stimulates their enthusiasm for classroom learning. Psychological film teaching, along with various art therapies and public art education, has a positive effect on mental health awareness. In practical applications, attention should be paid to individual differences, comprehensive application of various methods, and professional guidance to achieve the best results. Combining education with entertainment can stimulate student enthusiasm for classroom learning. Art therapy based on painting combines painting theory and psychological analysis, and has promising application prospects in mental health education. Expressive art therapy has a promising future in the field of group counseling for college students’ mental health (Zhang and Zhao, 2024b). Film-based intervention measures have been used in mental health education, and systematic evaluations indicate that film intervention has been accepted by teenagers as an educational tool, but its effectiveness in mental health education still needs further exploration (Liu et al., 2024).

#### 3.1.8 Enhancing the students’ psychological adaptability

To enhance the psychological adaptability of college students through film and television education, it is necessary to avoid choosing overly simplistic or vulgar films to prevent failure in achieving the educational goal (Liu et al., 2024). The teaching design should focus on student participation and interaction, guiding them to conduct in-depth thinking and discussions (Xie et al., 2023). The effect evaluation should focus on a comprehensive assessment of students’ psychological adaptability and can combine quantitative and qualitative evaluations to understand students’ understanding and application of psychological adaptation (Liu et al., 2024).

### 3.2 The specific implementation of film education or the integration of films with art therapy

#### 3.2.1 Applied in college mental health courses

The implementation of art therapy in university mental health courses involves multiple aspects such as enhancing students’ emotional expression abilities, relieving stress, and strengthening self-awareness. Art therapy encompasses various forms, such as painting, music, dance, and drama (Liu et al., 2024). The implementation of art therapy in university mental health courses is a systematic project that requires joint efforts of course designers, teachers, and students. Art therapy promotes the mental health of college students only through scientific course design, effective activity organization, and objective effect evaluation can the role of art therapy in promoting the mental health of college students be truly exerted (Zhang and Zhao, 2024).

#### 3.2.2 The effect of different types of film and television works on mental health education

Which types of film and television works are the most *ef*fective for mental health education is worth exploring. The applications of film and television works in mental health education mainly include feature films, animated films, documentaries, and short films. Different types of film and television have their own advantages in mental health education. Choosing appropriate films and combining professional guidance and art therapy can effectively promote the mental health of college students (Liu et al., 2024). Film production can combine other art therapies and combine film works with art therapy to more effectively promote the mental health of teenagers (Van Lith and Ettenberger, 2023). Future research can further explore the impact of different types of film and television work on the mental health of different adolescent groups, providing a more scientific basis for mental health education. Narrative films convey mental health knowledge and values through storytelling, whereas instructional films focus more on directly imparting knowledge and skills. The advantages of narrative films in psychological education lie in helping viewers to better understand the complexity and diversity of psychological problems, thereby reducing prejudice and discrimination against patients with psychological problems. Narrative films have a lasting impact on and promote mental health (Liu et al., 2024). By watching instructional films, students can acquire mental health knowledge and master coping skills, thereby improving their psychological adaptability. Although systematic evaluation studies have shown that film intervention has been accepted by teenagers as an educational tool, its effectiveness in mental health education still requires further exploration (Guo et al., 2020; Fàbregues et al., 2023). Before and after the film intervention, standardized psychological measurement tools such as the Self-Rating Anxiety Scale (SAS) and the Self-Rating Depression Scale (SDS) can be used to assess students’ emotional states and mental health levels. By comparing the measurement results before and after the intervention, the impact of the film intervention on students’ mental health can be understood.

#### 3.2.3 Evaluating the effectiveness of psychological film teaching activities

**FIGURE 2:**
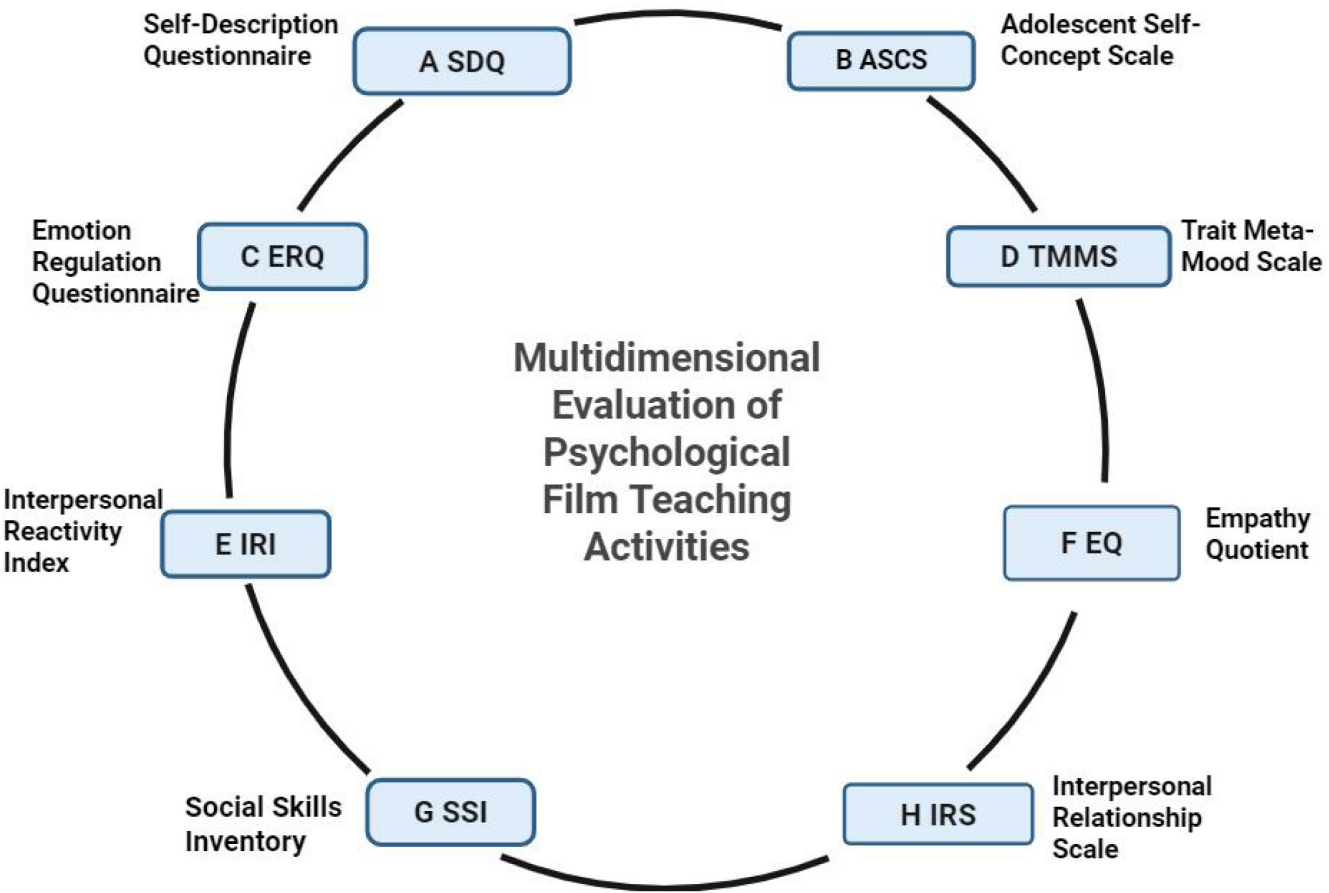
Overview of multidimensional assessment instruments for psychological film teaching activities. This figure displays eight questionnaires **(A–H)** used to evaluate various psychological outcomes related to film teaching interventions, covering self-perception, emotion regulation, empathy, social skills, and interpersonal relationships. The circular structure is used only to present the comprehensive and multidimensional nature of the evaluation system. It does not represent a fixed administration sequence, mandatory interconnection, or requirement to apply all scales simultaneously. Instead, this figure provides a clear summary of available assessment tools that can be selectively used according to specific research purposes. Figure created in BioRender.com.

The effectiveness of psychological film teaching activities can be evaluated in multiple dimensions. By comparing students’ test scores before and after the teaching activities, the improvement effect of the teaching activities on students’ mastery of psychological knowledge can be assessed (Müller et al., 2021; Zhang and Zhao, 2024b). This study employs a set of validated instruments, as summarized in Figures 2A–H, including the Self-Description Questionnaire (SDQ), Adolescent Self-Concept Scale (ASCS), Emotion Regulation Questionnaire (ERQ), Trait Meta-Mood Scale (TMMS), Interpersonal Reactivity Index (IRI), Empathy Quotient (EQ), Social Skills Inventory (SSI), and Interpersonal Relationship Scale (IRS), etc., (Liu et al., 2024; Marsh et al., 2005; Xu et al., 2020; de Lima and Osório, 2021). These tools correspond to distinct psychological constructs including self-perception, emotion regulation, empathy, social skills, and interpersonal relationships. While the circular layout may suggest a fixed sequence or mandatory use, it is intended to emphasize the multidimensional scope of the evaluation framework, allowing flexible selection of measures according to research needs. For example, the ERQ (Figure 2C) and IRI (Figure 2E) were specifically used to assess changes in emotion regulation and empathy. Collectively, these comprehensive assessments provide an objective evaluation of psychological film teaching and support the further optimization of instructional design.

## 4 Discussion

### 4.1 Film intervention reduces the stigmatization of psychological problems among college students

Film interventions may play an effective role in reducing stigmatization of mental problems among teenagers. As an innovative mental health education method, film intervention has the potential to reduce stigmatization of mental problems among students. Systematic reviews have shown that film intervention has been accepted by teenagers as an educational tool, but its effectiveness in mental health education still needs further exploration (Liu et al., 2024). Future research can further explore the effectiveness of film interventions in different cultural backgrounds and how to better utilize films to promote the mental health of teenagers.

#### 4.1.1 Challenges faced in psychological film teaching

Selecting an appropriate film is a primary challenge in psychological film teaching. When choosing a film, the cultural background of the students must be considered, as well as the basic concepts and principles of physiological psychology (Zhang and Zhao, 2024b; Liu et al., 2024). Teachers can guide students to analyze problems from different perspectives, thereby promoting their cognitive development and enhancing their ethical awareness (Tilden et al., 1994; Fan and Cui, 2024). Psychological film teaching faces many challenges in practical application, and it requires the joint efforts of teachers, students, and schools to continuously explore and improve so as to better play its role in mental health education.

#### 4.1.2 Therapeutic mechanism of film production

Film production provides college students with a safe, non-verbal channel for emotional expression (Krishnasamy and Van Lith, 2025b). During scriptwriting, scene design, and filming, students can externalize both negative emotions (e.g., anxiety, stress, depression) and positive emotions (e.g., joy, hope, gratitude) into visual narratives, enabling emotional catharsis and regulation without the constraints of verbal communication. Film production also represents an active process of self-exploration and identity construction (Goodwin et al., 2021; Tripon, 2024). By shaping characters and plots based on personal experiences and psychological needs, students can reexamine their self-identity, values, and life decisions throughout the creative process. For college students in a critical developmental stage of identity formation, this process supports the establishment of a stable and positive self-concept. Through narrative construction in film production, college students can assign new meaning to their lived experiences (Du et al., 2022). For those who have encountered academic setbacks, interpersonal conflicts, or other adverse events, filmmaking enables the reconstruction of negative life narratives, allowing individuals to identify positive implications within adversity and develop adaptive cognitive appraisals. This process effectively reduces psychological distress associated with challenging life events. Furthermore, film production is a collaborative creative activity (Black et al., 2025b; Popova and Nosek, 2025b). During shared brainstorming, filming, and editing, students engage in continuous communication, mutual cooperation, and social support. Such interactions help build and strengthen social support networks, enhance interpersonal skills, and alleviate loneliness and social anxiety, ultimately promoting improved social adaptation and mental health.

### 4.2 Future research directions (inferred from current trends)

**FIGURE 3:**
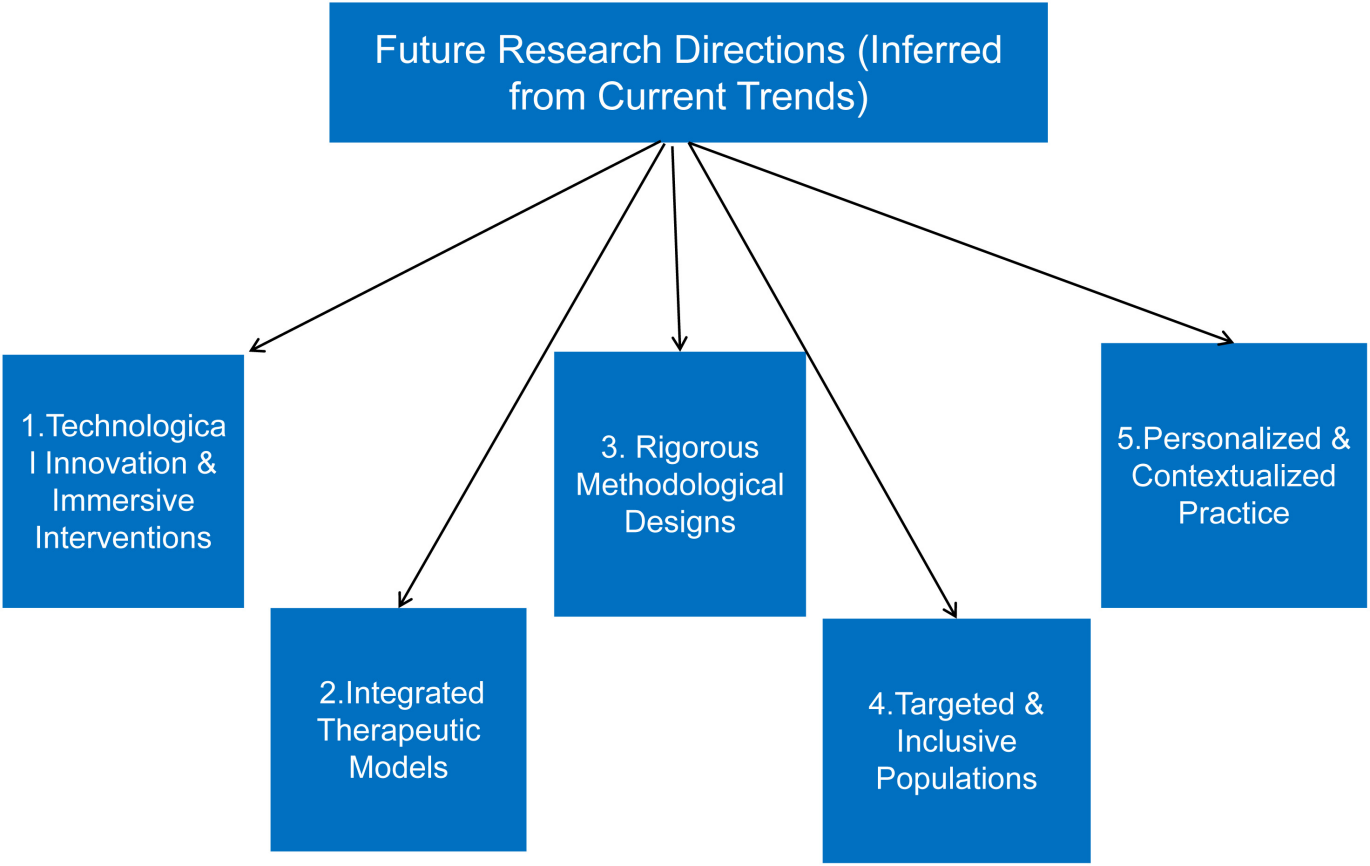
Future research directions in film-enhanced art therapy and mental health promotion for college students, as inferred from current trends and evidence. These directions are organized into five key domains: (1) technological innovation and immersive interventions, (2) integrated therapeutic models, (3) rigorous methodological designs, (4) targeted and inclusive populations, and (5) Personalized and contextualized practice.

Building on current evidence, future research in film education and art therapy for mental health should prioritize the following directions (Figure 3).

#### 4.2.1 Technological innovation and immersive interventions

Explore virtual art therapy by integrating virtual reality (VR) and augmented reality (AR) into film production, offering students more immersive and personalized therapeutic experiences (von Thienen et al., 2023). This includes leveraging AI-enhanced art therapy (AIATs), such as AI-generated art, chatbots, and robotic integration, to create novel, adaptive treatment approaches that enhance emotional expression and stress relief (Shamri Zeevi, 2021; von Thienen et al., 2023; Worsley et al., 2022; Hacmun et al., 2018).

#### 4.2.2 Integrated therapeutic models

Combine film production with evidence-based psychotherapies, such as cognitive behavioral art therapy (CBT-AT), to help students identify and reframe unhealthy cognitive and emotional patterns (Shamri Zeevi, 2021). Furthermore, art therapy is poised to place greater emphasis on integration with established psychotherapeutic approaches, such as cognitive behavioral therapy (CBT), while creative art therapy (CAT) has emerged as a prevalent and effective modality for advancing college students’ mental health (Luo et al., 2024). The therapeutic efficacy of such interventions can be further enhanced when combined with interdisciplinary strategies that incorporate family and community engagement (De Simone et al., 2025).

#### 4.2.3 Rigorous methodological designs

Employ more stringent experimental designs and quantitative methods to evaluate the specific impacts of film production on college students’ mental health and psychological resilience (Apolinário-Hagen et al., 2020; Luo et al., 2024). This includes using standardized psychological measurement tools, collecting objective behavioral data, and conducting long-term follow-up studies to assess the persistence of therapeutic effects and their influence on long-term student development (Fàbregues et al., 2023; Kubrak, 2020).

#### 4.2.4 Targeted and inclusive populations

Focus on specific student populations with distinct mental health needs, such as those with post-traumatic stress disorder (PTSD), to determine the effectiveness of film-based interventions (Ribeiro et al., 2021; Wang et al., 2025). Expand sample sizes and population scope, and conduct cross-cultural and multi-center studies to enhance the generalizability of findings across diverse contexts (Van Lith and Ettenberger, 2023; José et al., 2023).

#### 4.2.5 Personalized and contextualized practice

Emphasize personalization and specialization in art therapy, tailoring interventions to individual student needs (Wang et al., 2025). Further investigate the impact of different film types on diverse student groups and cultural backgrounds, and develop guidelines for selecting appropriate films and combining them with professional guidance to maximize therapeutic outcomes (Zhang and Zhao, 2024b; Hoover et al., 2022; Yu et al., 2022). Further research is warranted to elucidate the specific application modalities and therapeutic effects of the integration of film and art therapy, which will yield more evidence-based and effective strategies for mental health education and clinical intervention (Li L. et al., 2021; Decety and Meyer, 2008; McDonald et al., 2024; Li et al., 2025; Saravanapperumal and Ilamaparithi, 2025).

In summary, future research should aim to strengthen the empirical foundation of film education and art therapy by advancing technological integration, refining methodological rigor, and expanding inclusive, targeted applications. These efforts will broaden the prospects for using film-based interventions to promote mental health in educational and clinical settings.

### 4.3 Cluster analysis of key words regarding the research on the effects of art therapy on mental health

**FIGURE 4:**
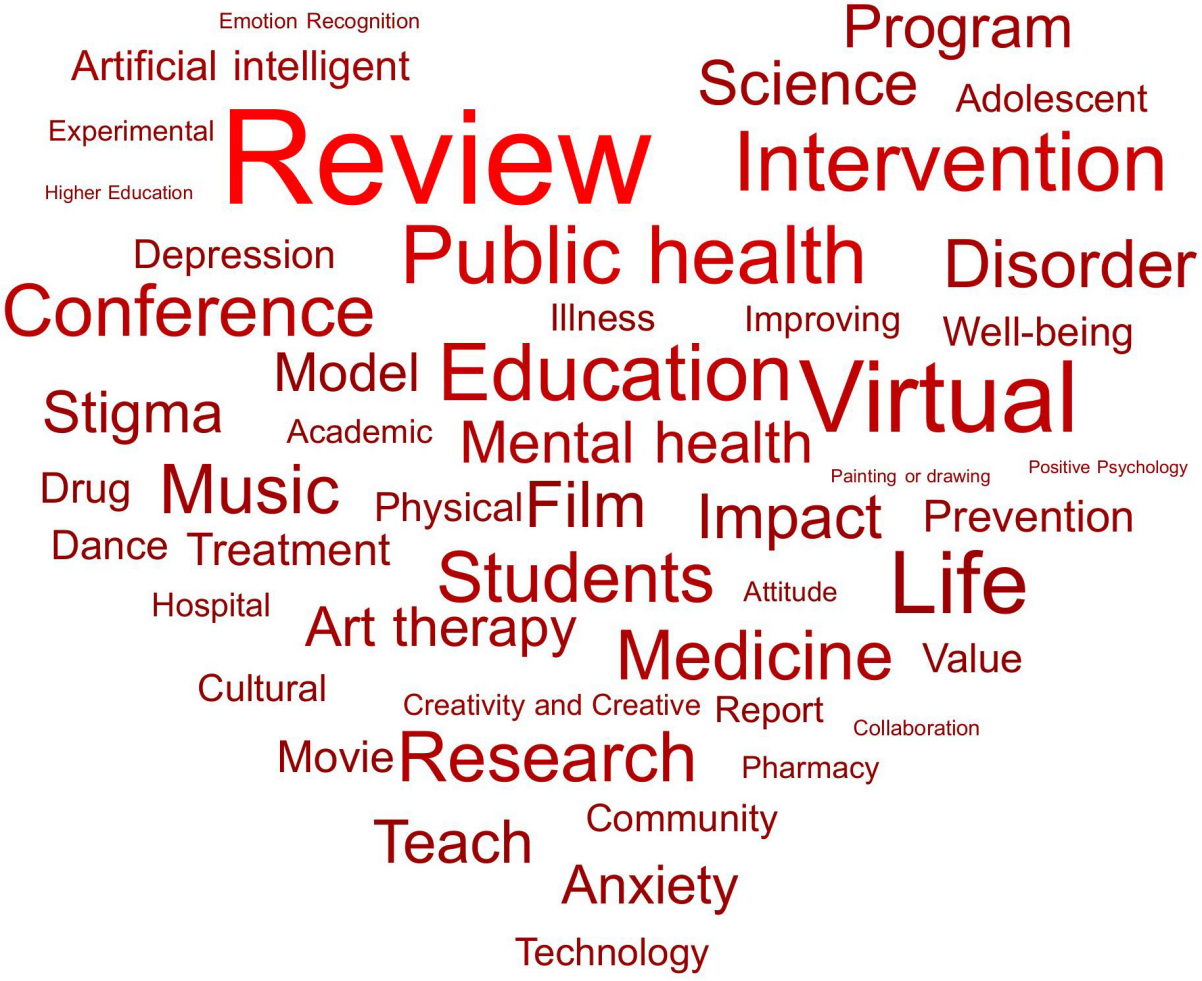
The Word cloud chart related to Film and art therapy on the mental health of college students data from PubMed 2020–2025.

On the PubMed platform, a search for papers (2020–2025) on the impact of art therapy including film and film education on mental health reveals a prominent and multifaceted connection between art and mental health support. Frequently appearing terms such as Review, Education, Public health, Intervention, and Virtual frame this field as evidence-based and evolving, while art therapy-related keywords like Music and Film and psychological terms such as Life, Stigma, Mental health, Disorder, and medical content highlight the diverse manifestations of art education in therapeutic contexts [Figure 4, visualized based on Supplementary Table 2 details the specific intervention measures of each study (such as film type, art therapy form, implementation frequency, etc.)]. These terms not only reflect practical responses to mental health challenges but also demonstrate growing public attention to art therapy’s role in mental wellbeing, with film emerging as a key medium that bridges mental health and education, enhancing its subjectivity, recognition, and influence in therapeutic practice.

This word cloud further crystallizes key themes at the intersection of film education, mental health, and therapeutic practice. Frequent terms including Review, Education, Mental health, Art therapy, Virtual, Students, Intervention, and Stigma collectively position film education as a multifaceted, evidence-based practice with significant therapeutic potential. As the most prominent term, Review underscores the critical role of systematic literature synthesis, critical analysis, and reflective practice in advancing film education: it enables the translation of research into evidence-based curricula, the evaluation of program effectiveness, and the identification of knowledge gaps to guide future inquiry. Terms like Mental health, Art therapy, and Intervention frame film production as a creative therapeutic medium one that fosters emotional expression, stress reduction, and psychological resilience through collaborative storytelling. Meanwhile, Virtual and Technology highlight the evolving accessibility of film-based interventions, while Students, Adolescent, and Higher Education emphasize the need to tailor these approaches to the developmental needs of young adults. Finally, Stigma and Public health underscore film education’s potential to destigmatize mental health and serve as a scalable public health strategy.

Recognizing Review as the most frequent term is particularly valuable: it confirms that film education and its therapeutic applications are rooted in rigorous, synthesized research, enhancing credibility for advocating film-based interventions in academic and clinical settings. It also provides actionable insights for curriculum design, guides impactful future research, and strengthens stakeholder communication ultimately supporting the integration of film education into education and healthcare systems to promote holistic student wellbeing. Looking ahead, this integration is poised to attract more individuals with psychological disorders, especially students, to engage in film appreciation and production, advancing related educational practices and fostering positive therapeutic outcomes for mental health development.

**FIGURE 5:**
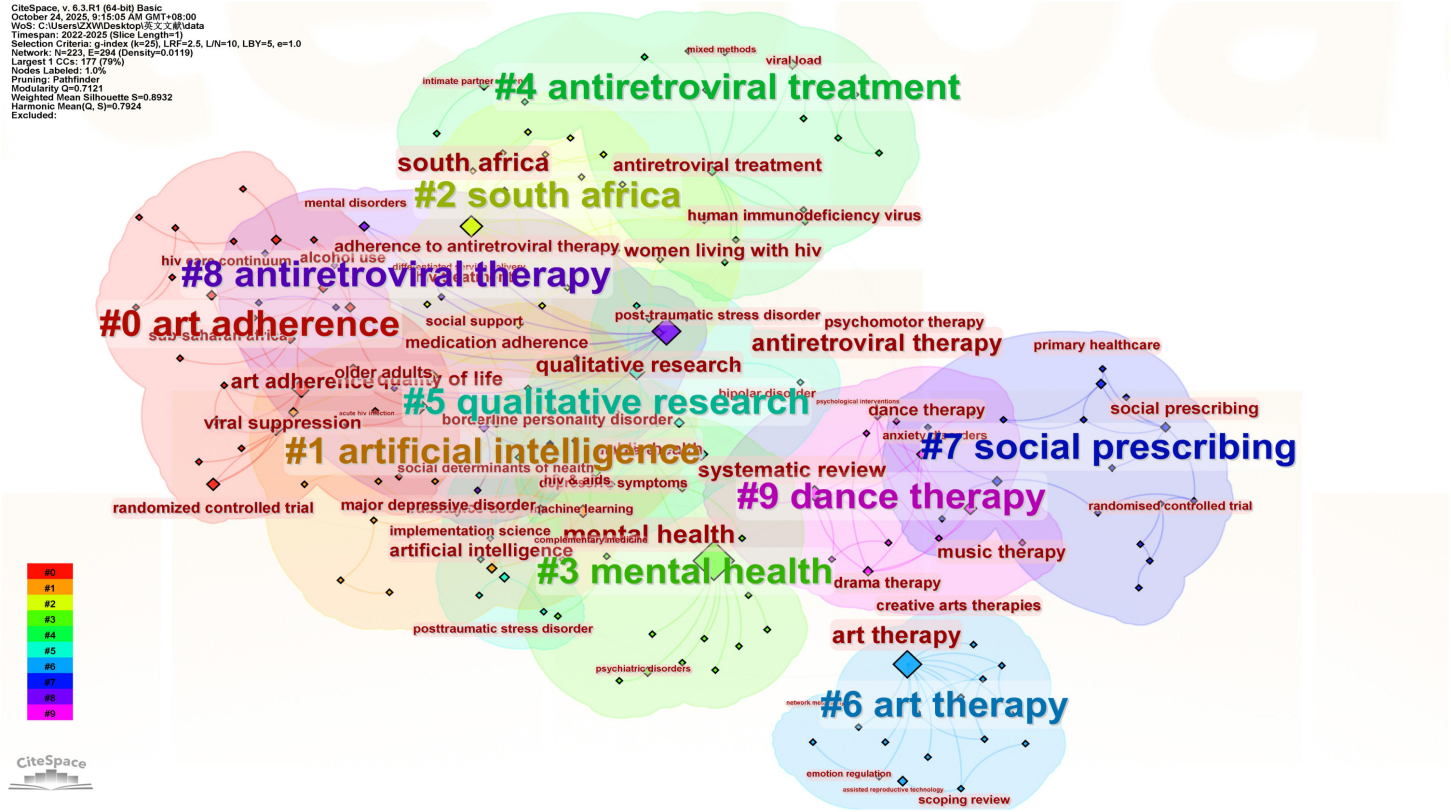
Visual analysis of art therapy on the mental health based on CiteSpace (data from PubMed to 2022–2025). The keyword timeline graph is plotted with time as the horizontal axis, keywords are divided according to the time units. Through the timeline graph, one can visually see the research hotspots within each time segment in this field and can comprehensively observe the changes in research hotspots across the time scale, grasping the latest developments and prospects of this field.

By combining Figure 5 [visualized based on Supplementary Table 3: PubMed Search Results (2020–2025); Supplementary Table 3 collates the outcome indicators and statistical results used in all studies, providing complete data support for the comprehensive analysis], we can see that the research hotspots in art therapy and mental health over the past three years have shown a dynamically changing pattern over time. And the keywords related to art such as “art adherence,” “art therapy,” and “dance therapy” gradually became the top priorities in this research field, and related targeted research such as “quality of life,” “Alternative therapy art in health” and “drama therapy” also gradually increased; at the same time, with the development of new technologies, such as “artificial intelligence,” “antiretroviral therapy or treatment,” “social prescribing,” and “qualitative research” began to appear frequently in mental health research and received widespread research attention.

From the visual analysis of the integration of film and art therapy in mental health research over the past 3 years, film and art therapy has become increasingly mature in its inward exploration with the support of production technologies. It also reflects the developmental trajectory and morphological changes in films and art therapy. Based on this, this article, combined with data analysis, draws research conclusions, conducts a flow change sort out of the developmental changes of film and art theory on mental health in recent years, and discusses the future development direction of film art therapy on mental health.

### 4.4 Limitations

This systematic review has several limitations that should be acknowledged. First, only one database (PubMed) was searched for literature retrieval, which may lead to potential omissions of relevant studies published in other databases. Second, the heterogeneity of included studies in terms of research design, intervention duration, and outcome measures may limit the comparability and generalizability of the synthesized results. Third, the number of eligible studies included in this review is relatively limited, which may reduce the robustness of the conclusions. These limitations should be considered when interpreting the findings of this review.

## 5 Conclusion

This systematic literature review focuses on the impact of film education and the integration of film and art therapy on the mental health and psychological resilience of college students within the context of film production education, aiming to explore how film production, as an art therapy approach, affects college students’ mental health and psychological resilience. Through a comprehensive analysis of existing research, this study achieves a more thorough understanding of the potential of film education and production in fostering college students’ mental health, offering a reference for future research and practice. Film production not only improves college students’ self-expression skills but also strengthens their social support networks and problem-solving capabilities, thereby comprehensively elevating their mental health levels. This review synthesizes current evidence on the application of art therapy and film education in promoting college students’ mental health, and its findings largely confirm the study hypothesis that the integration of film education and art therapy constitutes a meaningful, multidimensional approach to facilitating emotional expression, stress reduction, self-efficacy enhancement, and social support improvement. Film-based creative activities act as a safe and effective medium for psychological development, while art therapy supports emotional regulation and the cultivation of coping skills. Despite the identified limitations, the results validate the value of combining film education and art therapy in mental health intervention programs for university students. Future research is recommended to explore virtual art therapy integrated with cognitive behavioral therapy, adopt more rigorous experimental designs and quantitative methods, conduct long-term follow-up investigations, target specific populations, and implement comprehensive literature searches to further verify and expand these conclusions.
