# Supplementary material for: Film education and art therapy for mental health in college students: a systematic review
Source: Front Psychol. 2026 Apr 17;17:1749029. doi: 10.3389/fpsyg.2026.1749029 (PMC13133068; doi:10.3389/fpsyg.2026.1749029)
Supplement: Supplementary file 1 [file Supplementary_file_1.docx]

| **Section and Topic** | **Item #** | **Checklist item** | **Location where item is reported** |
| --- | --- | --- | --- |
| **TITLE** | | | Title page |
| Title | 1 | Identify the report as a systematic review. | Title: Film Education and Art Therapy for Mental Health in College Students: A Systematic Review |
| **ABSTRACT** | | | Abstract  section |
| Abstract | 2 | See the PRISMA 2020 for Abstracts checklist. | Abstract section(structured with Objectives, Methods, Results, Conclusions) |
| **INTRODUCTION** | | | Introduction  section |
| Rationale | 3 | Describe the rationale for the review in the context of existing knowledge. | Introduction (1st paragraph) |
| Objectives | 4 | Provide an explicit statement of the objective(s) or question(s) the review addresses. | Introduction (2nd and 3rd paragraph) |
| **METHODS** | | | Methods section (all subsections) |
| Eligibility criteria | 5 | Specify the inclusion and exclusion criteria for the review and how studies were grouped for the syntheses. | Methods 2.1 Eligibility Criteria |
| Information sources | 6 | Specify all databases, registers, websites, organisations, reference lists and other sources searched or consulted to identify studies. Specify the date when each source was last searched or consulted. | Methods 2.1 Information Sources (PubMed; last searched: 2025.02.12; time range:2020.01.01-2025.12.31) |
| Search strategy | 7 | Present the full search strategies for all databases, registers and websites, including any filters and limits used. | Methods 2.1 Search Strategy ((art therapy) AND (film) AND (mental health); filters: human studies, peer-reviewed full-text) |
| Selection process | 8 | Specify the methods used to decide whether a study met the inclusion criteria of the review, including how many reviewers screened each record and each report retrieved, whether they worked independently, and if applicable, details of automation tools used in the process. | Methods 2.2 Study Selection and Data Extraction (2 independent reviewers; third reviewer for dispute resolution; no automation tools) |
| Data collection process | 9 | Specify the methods used to collect data from reports, including how many reviewers collected data from each report, whether they worked independently, any processes for obtaining or confirming data from study investigators, and if applicable, details of automation tools used in the process. | Extraction (2 independent reviewers; third reviewer for dispute resolution; no automation tools) |
| Data items | 10a | List and define all outcomes for which data were sought. Specify whether all results that were compatible with each outcome domain in each study were sought (e.g. for all measures, time points, analyses), and if not, the methods used to decide which results to collect. | Methods 2.2 Study Selection and Data Extraction (Outcomes: anxiety/depression symptoms, emotional regulation, quality of life, social function; all time points/measures collected) |
|  | 10b | List and define all other variables for which data were sought (e.g. participant and intervention characteristics, funding sources). Describe any assumptions made about any missing or unclear information. | Methods 2.3 Study Selection and Data Extraction (Variables: study design, population, intervention duration; no missing/unclear information assumed) |
| Study risk of bias assessment | 11 | Specify the methods used to assess risk of bias in the included studies, including details of the tool(s) used, how many reviewers assessed each study and whether they worked independently, and if applicable, details of automation tools used in the process. | Methods 2.4 Risk of Bias Assessment (Cochrane RoB 2 for RCTs, NOS for observational studies; 2 independent reviewers; three automation tools) |
| Effect measures | 12 | Specify for each outcome the effect measure(s) (e.g. risk ratio, mean difference) used in the synthesis or presentation of results. | Results 3 Main Outcomes (descriptive efficacy rates) |
| Synthesis methods | 13a | Describe the processes used to decide which studies were eligible for each synthesis (e.g. tabulating the study intervention characteristics and comparing against the planned groups for each synthesis (item #5)). | Methods 2.4 Synthesis Methods (all eligible studies included for qualitative synthesis; grouped by intervention duration/format) |
|  | 13b | Describe any methods required to prepare the data for presentation or synthesis, such as handling of missing summary statistics, or data conversions. | Methods 2.4 Synthesis Methods (no missing statistics; no data conversions performed) |
|  | 13c | Describe any methods used to tabulate or visually display results of individual studies and syntheses. | Results 3.1 Study Selection (PRISMA flow diagram); Results 3.2/3.3 (narrative tabulation of study characteristics/outcomes) |
|  | 13d | Describe any methods used to synthesize results and provide a rationale for the choice(s). If meta-analysis was performed, describe the model(s), method(s) to identify the presence and extent of statistical heterogeneity, and software package(s) used. | Methods 2.4 Synthesis Methods (narrative qualitative synthesis; rationale: high study design/population heterogeneity; no meta-analysis) |
|  | 13e | Describe any methods used to explore possible causes of heterogeneity among study results (e.g. subgroup analysis, meta-regression). | Methods 2.4 Synthesis Methods / Results 3 (subgroup analysis by intervention duration (≥3m/<3m) and format (group/individual)) |
|  | 13f | Describe any sensitivity analyses conducted to assess robustness of the synthesized results. | Methods 2.4 Synthesis Methods (no sensitivity analyses conducted) |
| Reporting bias assessment | 14 | Describe any methods used to assess risk of bias due to missing results in a synthesis (arising from reporting biases). | Discussion (no formal assessment; minimal reporting bias as full-text peer-reviewed studies included) |
| Certainty assessment | 15 | Describe any methods used to assess certainty (or confidence) in the body of evidence for an outcome. | Methods 2.4 Synthesis Methods / Results 3(GRADE method used for evidence certainty assessment) |
| **RESULTS** | | | Results section (all subsections) |
| Study selection | 16a | Describe the results of the search and selection process, from the number of records identified in the search to the number of studies included in the review, ideally using a flow diagram. | Results 3.1 Study Selection (PRISMA 2020 flow diagram with full numerical breakdown) |
|  | 16b | Cite studies that might appear to meet the inclusion criteria, but which were excluded, and explain why they were excluded. | Results 3.1 Study Selection (exclusion reasons detailed in flow diagram; no individual study citation for excluded full-texts) |
| Study characteristics | 17 | Cite each included study and present its characteristics. | Results 3.1 Characteristics of Included Studies (narrative summary of all 22 studies; study design/population/intervention duration) |
| Risk of bias in studies | 18 | Present assessments of risk of bias for each included study. | Results 3 Risk of Bias (narrative summary; most studies low-moderate risk of bias) |
| Results of individual studies | 19 | For all outcomes, present, for each study: (a) summary statistics for each group (where appropriate) and (b) an effect estimate and its precision (e.g. confidence/credible interval), ideally using structured tables or plots. | Results 3 Main Outcomes (narrative summary of aggregate statistics/effect estimates for all studies) |
| Results of syntheses | 20a | For each synthesis, briefly summarise the characteristics and risk of bias among contributing studies. | Results 3 Main Outcomes / Risk of Bias (all 22 studies included; low-moderate overall risk of bias) |
|  | 20b | Present results of all statistical syntheses conducted. If meta-analysis was done, present for each the summary estimate and its precision (e.g. confidence/credible interval) and measures of statistical heterogeneity. If comparing groups, describe the direction of the effect. | Results 3 Main Outcomes (81.8% studies showed significant positive effect; long-term/group interventions superior to short-term/individual) |
|  | 20c | Present results of all investigations of possible causes of heterogeneity among study results. | Results 3 Main Outcomes (heterogeneity causes: intervention protocol standardization, population baseline mental health status) |
|  | 20d | Present results of all sensitivity analyses conducted to assess the robustness of the synthesized results. | Results 3 Main Outcomes (no sensitivity analyses conducted) |
| Reporting biases | 21 | Present assessments of risk of bias due to missing results (arising from reporting biases) for each synthesis assessed. | Discussion (minimal reporting bias; no quantitative assessment) |
| Certainty of evidence | 22 | Present assessments of certainty (or confidence) in the body of evidence for each outcome assessed. | Results 3 Risk of Bias / Discussion(moderate certainty for primary outcomes; high certainty for intervention feasibility) |
| **DISCUSSION** | | | Discussion section (all paragraphs) |
| Discussion | 23a | Provide a general interpretation of the results in the context of other evidence. | Discussion (consistent with the evidence on art therapy efficacy) |
|  | 23b | Discuss any limitations of the evidence included in the review. | Discussion 4.4 Limitations(high intervention heterogeneity, limited studies) |
|  | 23c | Discuss any limitations of the review processes used. | Discussion 4.4 Limitations (single database search (PubMed), no grey literature included) |
|  | 23d | Discuss implications of the results for practice, policy, and future research. | Discussion 4.2(clinical practice: adjuvant therapy recommendation; future research: standardized protocols, large-sample RCTs) |
| **OTHER INFORMATION** | | | Title page / Acknowledgements/ Endnote |
| Registration and protocol | 24a | Provide registration information for the review, including register name and registration number, or state that the review was not registered. | Title page (PROSPERO; CRD42025030188) |
|  | 24b | Indicate where the review protocol can be accessed, or state that a protocol was not prepared. | Endnote (protocol available on PROSPERO website) |
|  | 24c | Describe and explain any amendments to information provided at registration or in the protocol. | Endnote (no amendments to registered protocol) |
| Support | 25 | Describe sources of financial or non-financial support for the review, and the role of the funders or sponsors in the review. | Title page / Acknowledgements (no financial/non-financial support; independent review) |
| Competing interests | 26 | Declare any competing interests of review authors. | Title page / Acknowledgements (no competing interests declared by all authors) |
| Availability of data, code and other materials | 27 | Report which of the following are publicly available and where they can be found: template data collection forms; data extracted from included studies; data used for all analyses; analytic code; any other materials used in the review. | Endnote (all materials (data extraction form, study data) available upon reasonable request from corresponding author; no analytic code (no meta-analysis)) |

*From:*  Page MJ, McKenzie JE, Bossuyt PM, Boutron I, Hoffmann TC, Mulrow CD, et al. The PRISMA 2020 statement: an updated guideline for reporting systematic reviews. BMJ 2021;372:n71. doi: 10.1136/bmj.n71. This work is licensed under CC BY 4.0. To view a copy of this license, visit <https://creativecommons.org/licenses/by/4.0/>
