# Supplementary material for: Film education and art therapy for mental health in college students: a systematic review
Source: Front Psychol. 2026 Apr 17;17:1749029. doi: 10.3389/fpsyg.2026.1749029 (PMC13133068; doi:10.3389/fpsyg.2026.1749029)
Supplement: Supplementary file 2 [file Supplementary_file_2.docx]

**Identification of studies via databases and registers**

Records removed *before screening*:(n=0)

Records identified from:

PubMed (n=128)

Registers (n=0)

**Identification**

Records excluded:(n=89);

Irrelevant topic (n=52); Conference proceedings (n=21); Comments/editorials (n=16)；Reports not retrieved (n=0)

Records screened:(n=128)

Full-text articles excluded, with reasons:(n=11);

Incomplete data (n=4);

Ineligible study design (n=4); Population/intervention mismatch (n=3)

Reports assessed for eligibility:(n=39)

**Screening**

Studies included in qualitative synthesis:(n=28)

Studies included in qualitative synthesis:(n=28)

Studies included in quantitative synthesis (meta-analysis):(n=0)

**Included**

Source: Page MJ, et al. BMJ 2021;372:n71. doi: 10.1136/bmj.n71.

This work is licensed under CC BY 4.0. To view a copy of this license, visit <https://creativecommons.org/licenses/by/4.0/>
